# Cholera -“Rice water stools

**DOI:** 10.11604/pamj.2017.26.147.11874

**Published:** 2017-03-14

**Authors:** Lykourgos Christos Alexakis

**Affiliations:** 1 Médecins Sans Frontières, Athens, Greece; 2 1^st^ Department of Internal Medicine, “G.Gennimatas” General Hospital, Athens, Greece; 3 Aide Médicale Internationale, Mae Sot, Tak, Thailand

**Keywords:** Cholera, diarrhea, rice water stools

## Image in medicine

An adult female presented with acute watery diarrhoea and severe dehydration during a cholera outbreak in Mae La refugee camp, Thailand in 2007. Her stools had the classic appearance of cholera “rice water stools”, which is similar to water where rice was boiled. Stool are watery, greyish in colour, cloudy, with flecks of mucus, with a non offensive fish-like odour (Panels A, B, C and D from this patient). The patient was isolated, treated initially with Ringers’s lactate intravenously and then with oral rehydration solution. Tetracycline per os was given for three days according to local protocol and the patient recovered completely. A culture of stools obtained by rectal swab upon admission subsequently tested positive for Vibrio Cholerae, 01 Inaba serotype, which was also the cause of the outbreak. Cholera is transmitted through contaminated food or water, can cause epidemics and may also infect travellers. Clinical spectrum ranges from asymptomatic to severe disease with massive watery diarrhoea often fatal if untreated. Treatment includes rapid fluid replacement with the WHO’s reduced osmolarity oral rehydration solution or intravenous rehydration (Ringer’s lactate) for severe cases. Antibiotics (azithromycin, erythromycin,tetracycline or ciprofloxacine) decrease disease duration.

**Figure 1:**
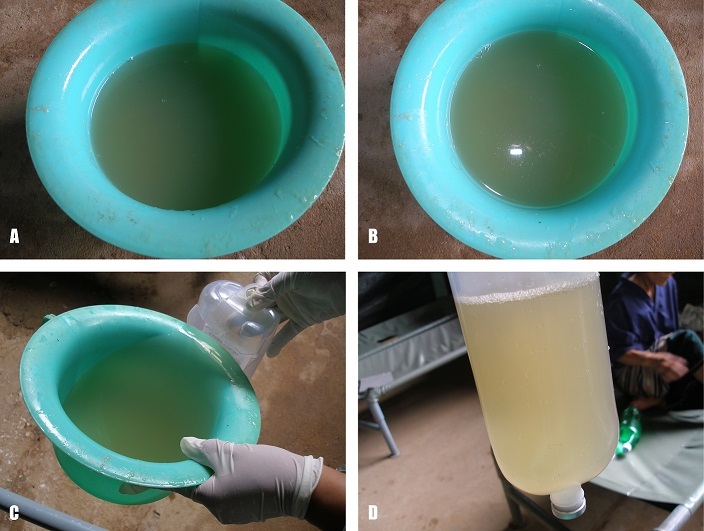
A) cholera “rice water” stools; B) flecks of mucus visible on the surface; C) the watery quality can be seen during transport into a transparent container; D) cholera stools are greyish, cloudy, similar to water where rice was boiled

